# Cyanidin-3-Glucoside Modulates hsa_circ_0001345/miRNA106b/ATG16L1 Axis Expression as a Potential Protective Mechanism against Hepatocellular Carcinoma

**DOI:** 10.3390/cimb44040115

**Published:** 2022-04-12

**Authors:** Shaimaa Zabady, Nievin Mahran, Mohamed A. Soltan, Muhammad Alaa Eldeen, Refaat A. Eid, Sarah Albogami, Eman Fayad, Marwa Matboli, Eman K. Habib, Amany H. Hasanin, Mahmoud A. Ali, Noha M. Mesbah, Dina M. Abo-Elmatty, Asmaa R. Abdel-Hamed

**Affiliations:** 1 Department of Biochemistry, Faculty of Pharmacy, Sinai University, Ismailia 16020, Egypt; shaimaazabady38@gmail.com; 2 Department of Biochemistry, Faculty of Dentistry, Sinai University, Ismailia 16020, Egypt; nievin.mahran@su.edu.eg; 3 Department of Microbiology and Immunology, Faculty of Pharmacy, Sinai University, Ismailia 16020, Egypt; 4 Cell Biology, Histology & Genetics Division, Zoology Department, Faculty of Science, Zagazig University, Alsharquia 44519, Egypt; 5 Department of Pathology, College of Medicine, King Khalid University, Abha 12573, Saudi Arabia; refaat_eid@yahoo.com; 6 Department of Biotechnology, College of Science, Taif University, Taif 21944, Saudi Arabia; dr.sarah@tu.edu.sa (S.A.); e.esmail@tu.edu.sa (E.F.); 7 Department of Medical Biochemistry and Molecular Biology, Faculty of Medicine, Ain Shams University, Cairo 11566, Egypt; marwasayed472@yahoo.com; 8 Faculty of Medicine, Galala University, Galala City 43511, Egypt; emanhabib75@gmail.com; 9 Department of Anatomy, Faculty of Medicine, Ain Shams University, Cairo 11566, Egypt; 10 Department of Clinical Pharmacology, Faculty of Medicine, Ain Shams University, Cairo 11566, Egypt; helmy_amany@yahoo.com; 11 Department of Molecular Microbiology, Faculty of Medicine, Armed Forces College, Cairo 11566, Egypt; mahmoudali@gmail.com; 12 Department of Biochemistry, Faculty of Pharmacy, Suez Canal University, Ismailia 41522, Egypt; noha_mesbah@pharm.suez.edu.eg (N.M.M.); dina_abouelmouti@pharm.suez.edu.eg (D.M.A.-E.); asmaa.ramdan@pharm.suez.edu.eg (A.R.A.-H.)

**Keywords:** autophagy, hepatocellular carcinoma, cyanidin-3-glucoside, circular RNA, micro RNA

## Abstract

Hepatocellular carcinoma (HCC) is the most common form of malignancy in the liver. Autophagy was found to have a significant effect in controlling HCC. Anthocyanins, which are naturally occurring pigments in a variety of fruits and vegetables, have been thoroughly documented to be involved in a variety of bioactive activities and are widely employed for their antioxidant capabilities. Cyanidin-3-glucoside (C3G) extracted from *Morus alba* L. has promising antioxidant and anti-tumour activities. The current study aims to examine the protective action of C3G against hepatocellular carcinoma through the investigation of the autophagy protein ATG16L1 expression along with its related RNA molecules (hsa_circ_0001345 and miRNA106b) in Wistar rats. In vivo precancerous lesions (PCL) were induced using diethylnitrosamine (DEN) and acetamidofluorene (2-AAF). Rats were treated with C3G (10, 15, and 20 mg/kg; 4 times weekly) for 112 days (16 weeks). Liver function tests, alfa fetoprotein, ATG16L1 expression, hsa_circ_0001345, and miRNA106b differential expression were examined. Liver sections were examined by histological and immunohistochemical approaches. The current study’s findings indicated that C3G administration protects against the negative effects of DEN-2-AAF on liver functions and liver histopathological sections, which nominated C3G as a potential prophylactic agent against HCC.

## 1. Introduction

Hepatocellular carcinoma (HCC) is considered a major cause of death in cancer suffering patients globally. Although there have been massive advances in diagnosis and treatment techniques in the last few years, the incidence and mortality of HCC are still rising [1]. Many factors have been attributed to HCC, with hepatitis C and B at the top of the list [2]. In Egypt, a country with a high prevalence of hepatitis C virus, the occurrence of HCC increased from 1.4 to 6.2 per 100,000 individuals in the last 30 years [3]. Resistance to chemotherapy is a crucial aspect to take into consideration while treating HCC cases. The principal resistance mechanisms against traditional anti-tumour medicines included several factors, such as decreased drug absorption, increased efflux rate, and altering cell survival signalling, as well as suppression of the apoptotic machinery [4].

Since ancient history, *Morus alba* L. fruits (mulberry) have been recognised as a healthful food and commonly consumed in Asian and European countries, such as Korea, China, Serbia, and Japan. Recent studies have suggested that cyanidin-3-glucoside (C3G), an anthocyanin flavonoid isolated from mulberry fruit, has been demonstrated to significantly reduce oxidative stress [5], enhance the lipid profile [6], and have ameliorative impacts in animal models of different disorders [7]. Standardised anthocyanins contain a very large amount of polyphenols and exhibit comparable benefits [8]. Current research has established the critical role of anthocyanins as a dietary antioxidant in preventing oxidative damage, inhibiting cancer cell proliferation, and modulating cell development in vitro [9]. However, the molecular mechanisms underlying mulberry cyanidin-3-glucoside’s anticancer effects remain controversial.

In cancer research, C3G was studied against several cancer models. It inhibits ethanol-induced invasion of breast cancer [10] and migration of lung cancer [11], induces apoptosis, and suppresses tumour growth [12]. Moreover, C3G can protect against ultraviolet-induced injury through up-regulating autophagy [13].

Autophagy is a vital process done regularly by normal cells through lysosomal degradation to maintain cellular homeostasis under basal and stressed states [14]. It runs on consecutive stages from initiation to elongation and finally the maturation stage, where several cellular components are incorporated in this process [15]. Regarding tumour progression, specifically for HCC, autophagy was largely examined for its role in fighting malignant cells by promoting apoptosis [16]. Understandably, autophagy deficiency increases the damaged mitochondria, resulting in oxidative stress that suppresses DNA repair and increases the mutation rate, the key factor for tumorigenesis [17].

Circular RNAs (circRNAs) are a specialised class of RNA due to the high stability of their covalently closed structures [18]. These non-coding RNA molecules have been studied for their diverse roles in several cellular processes [19]. Micro RNAs (miRNAs) are short non-coding RNA that have a vital role in the post-transcriptional regulation of gene expression [20]. About twenty years ago, the connection between cancer and miRNA was first discovered [21], and since then, miRNA molecules have been extensively studied under cancer development and diagnosis. Connections between circRNA and miRNA were investigated as the following: circRNAs can act as a microRNA sponge resulting in a regulation of specific gene expression. Consequently, tumour inhibition or activation would be anticipated.

In this study, we aim to examine the potential role of C3G as a prophylactic or protective agent against HCC through an in vivo study. The anti-tumour action of C3G was investigated by employing the liver function tests, immunopathological procedures, and estimation of a novel RNA axis that was selected to assess the improvement of the autophagy process following C3G administration.

## 2. Materials and Methods

### 2.1. Chemicals

Diethylnitrosamine (DEN) with a purity ≥99.0% and acetamidofluorene (2-AAF) with a purity ≥ 98% were obtained from Sigma Aldrich (St. Louis, MO, USA). C3G was purchased from Earth Natural Supplements-Colorado Spring-USA.

### 2.2. Experimental Animals

Forty male Wistar rats weighing 200–250 g were selected for the current study. The rats were housed under conditions of 22–24 °C, 12 h light–dark cycles, and fed on standard rat chow and tap water. A week adaptation period was provided before the start of the experimental procedures. The weight of each rat was measured to calculate the exact dosage of DEN and 2-AAF.

### 2.3. Experimental Protocol

The overall time of the experiment was 112 days (16 weeks). Rats were divided into five groups. Group 1 (six rats), which was injected with [0.9% (*w*/*v*) NaCl intraperitoneally; DEN vehicle], represented the normal control group. The remaining four groups were induced with precancerous lesions (PCL) by intraperitoneal injection (i.p.) of DEN + 2AAF. Regarding DEN, it was injected as 100 mg/kg once weekly for three consecutive weeks followed by a 1-week rest period. Following that, 2AAF was injected, i.p. with a dose of 300 mg/kg for one time. Group 2 was the PCL group (10 rats); rats orally received NaCl which was the vehicle for C3G, and was administered with the same protocol as C3G. Groups 3, 4, and 5 (T1, T2, and T3, respectively; 8 rats/group) were treated orally from the first day of the experiment with C3G four times weekly for 112 days (16 weeks) in doses 10 mg/kg, 15 mg/kg, and 20 mg/kg, respectively [22].

### 2.4. Collection of Blood and Liver Tissue

In order to collect the blood and liver tissue for analysis, rats were anesthetised with urethane (1.2 g/kg; i.p.) dissolved in distilled water, and retro-orbital blood samples were taken. Rats were sacrificed to collect the liver samples for histopathological and immunohistochemical studies. A portion of the liver was immediately fixed in buffered formalin (10% *v*/*v*) for 24 h for this purpose, and the other portion was kept at −80 °C for RNA extraction. Blood samples were centrifuged at 7000 rpm for 15 min to collect serum samples for liver function tests and stored at −20 °C.

### 2.5. Alfa Fetoprotein and Liver Function Tests

Alfa fetoprotein (AFP) was analysed by ELISA kit (AbCam cat no [ab108838], Cambridge, MA, USA). Alanine transaminase (ALT), albumin, total bilirubin (T bilirubin), and direct bilirubin (D bilirubin) were analysed with Beckman Coulter AU analysers (Krarmer Blvd, Brea, CA, USA).

### 2.6. In Silico Selection of Targeted circRNA-miRNA and Liver RNA Axis Analysis through RT-PCR

Human autophagy gene ATG16L1 was essential for the cellular autophagy process [23]. The circRNA-miRNA network related to ATG16L1 expression was identified from the circ2 trait database [24]. Pathway enrichment analysis was performed to ensure that the selected miRNA is linked to carcinogenesis via the Diana Tools database [25].

The extraction of liver tissue total RNA was performed with a Qiagen miRNeasy Mini Kit, and the concentration of the extracted RNA was detected by measuring absorbance using an Ultraspec 1000 (UV/visible spectrophotometer) where the ratio A260/A280 was detected. It was found to be from 1.8 to 2. Afterwards, reverse transcription to complementary DNA (cDNA) was performed with miScript II RT Kit and oligo-dT primers. RT reactions were carried out at 37 °C for an hour, then inactivated at 95 °C for 5 min. The reactions were adjusted to the volume of 10 μL and consisted of 2 μL 5× miScript Hiflex buffer, 1 μL 10× miScript nucleic mix, 1 μL RNase-free water, one μL miScript reverse transcriptase mix, and 5 μL RNA (70 μg/mL).

Quantitative PCR was performed with the Hs_miR-106b_1 miScript primer assay (cat no: 218300, ID: MS00003402) and the miScript SYBR Green PCR Kit, cat no: 218073; (Qiagen, Hilden, Germany). The reaction consisted of 10 μL; 5 μL miScript SYBR Green PCR master mix, 1 μL 10× miScript primer assay, 1 μL 10× miScript Universal primer, 2.5 μL RNase-free water, and 0.5 μL cDNA. Gene expression was normalised to the U6 gene, using the Hs_RNU6-2_11 miScript Primer Assay. The CircInteractome database was used to obtain the junction sequence of the targeted circRNA [26], and it was amplified with a custom design primer assay obtained from the primer-blast database [27]. ATG16L1 mRNA expression was measured with a QuantiTect Primer Assay and QuantiTect SYBR Green PCR Kit. QT-PCR reactions were carried out to the final volume of 10 μL, the volumes of added components were 5 μL, 0.5 μL, 0.5 μL, 3.5 μL, and 0.5 μL of QuantiTect SYBR Green PCR master mix, 10× primer A, 10× primer B, RNase-free water, and cDNA, respectively. Gene expression was normalised to the ACTB gene. RT- PCR was run with the cycling conditions of 95 °C for 15 min, 40 cycles of 94 °C for 15 s, 55 °C for 30 s, and 70 °C for 30 s. The comparative cycle threshold (ΔCt) method was applied to estimate miRNA expression where the mean Ct values were detected for the samples, then Ct values of the endogenous control were subtracted from Ct values of the target miRNA to obtain the ΔCt value. The standard 2^−ΔΔCt^ method was applied to assess the relative expression of miRNA [28]. Data are presented as fold change in competing endogenous RNAs (ceRNA) expression [29].

### 2.7. Histopathological and Immunohistochemical Studies

After fixation, liver slices (3–4 mm) were dehydrated and embedded in paraffin blocks. At least four cross-sections (5-μm thick) were taken from each and stained with hematoxylin and eosin (Hx & E). Microphotographs were taken with an Olympus BX50 microscope. For immunohistochemistry, tissues were dewaxed with xylene and hydrated by a graded ethanol series. Sections were incubated with 3% (*v*/*v*) H_2_O_2_ for 15 min to suppress endogenous peroxidase activity. Following that, the sections were gently washed with tap water, then rinsed with PBS (pH 7.3) and incubated with glutathione S-transferase P (GST-P) antibodies (0.5–1 μg/mg) overnight at 4 °C. Sections were rinsed with PBS and incubated with rabbit polyclonal antibody against GST-P diluted 1/250 overnight at 4 °C [30]. Then the sections were incubated with biotinylated 2ryAb for 30 min. GST-P was purchased from Abcam (San Francisco, CA, USA). The peroxidase activity was developed with 3,3′-Diaminobenzidine (DAB) solution. The protein of interest was targeted by an antibody that reacts with peroxidase in the presence of hydrogen peroxide; DAB is converted to its oxidised form, forming a brown precipitate. Counterstaining was performed with hematoxylin. All rats’ liver tissues in each group were examined by light microscope in five random fields.

### 2.8. Statistical Analysis

Data are expressed as mean ± standard deviation (SD). Differences in the relative expression of circRNA associated ceRNA were analysed by the one-way analysis of variance test (ANOVA). A *p*-value < 0.05 was considered statistically significant. Data were processed using the SPSS 20.0 software package.

## 3. Results

The death rate in the PCL group was 40%, while in the C3G treated group, it was 25%, 25%, and 12.5% in T1, T2, T3, respectively, with a 0% death rate in the normal control group.

### 3.1. AFP Measurement and Liver Function Test Results

Generally, the administration of C3G protects against the deterioration of the liver function tests. Serum ALT, T bilirubin, and D bilirubin showed a significant increase in the PCL group compared to the normal control group. It decreased significantly in rats treated with C3G in a dose-dependent manner compared to the PCL group (Table 1). Furthermore, serum albumin, significantly decreased by DEN + 2AAF injection compared to the normal control group, exhibited a significant increase in groups treated with C3G compared to the PCL group (Table 1). AFP level was increased significantly in the PCL group compared to the healthy normal control group. In contrast, groups treated with C3G showed a significant decrease in AFP levels compared to the same PCL group (Table 1).

### 3.2. Effect of Cyanidin-3-Glucoside on the Expression of (hsa_circ_0001345/miRNA106b/ATG16L1) Axis

The in silico analysis predicted ATG16L1 mRNA as a target of miRNA-106b through the Diana database analysis. Moreover, circRNA0001345/miRNA106b/ATG16L1 was estimated to be the main RNA axis for assessing C3G action on HCC. CircRNA0001345 was amplified with a custom design primer assay (forward primer 5′-TGTATCTCTACCCAAGCCCCA-3′ and reverse primer 5′-TCCCAAGCACATCTACGCAA-3′), obtained from the primer-blast database. circRNA0001345/miRNA106b/ATG16L1 expressions were estimated in liver tissues of the five groups.

CircularRNA0001345 expression was up-regulated in rats treated with C3G with the highest fold change value (23.37) in the T3 group compared to the PCL group (Table 2) while miRNA-106b expression was up-regulated in the PCL group (22.34 RQ) and did not show upregulation in treatment with different doses of C3G (RQs: 0.6, 0.23 and 0.21) respectively (Table 2). Expression of ATG16L1 was up-regulated in treated groups in a dose-dependent manner compared to the PCL group (Table 2).

### 3.3. Investigated RNA Axis in Liver Tissues

**Figure 1:**
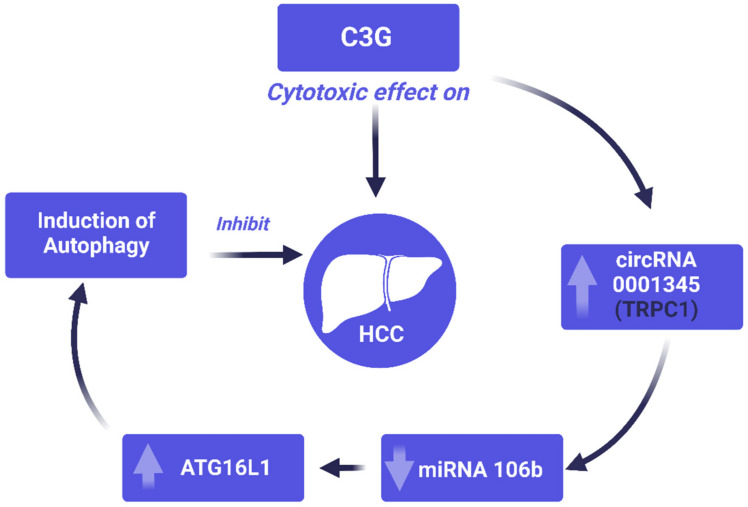
A schematic representation of the proposed pathway for the anticancer effect of C3G on modulating (circRNA0001345/miRNA106b/ATG16L1) axis.

Analysis of the current study axis of interest exhibited a significant positive correlation between circ-RNA0001345 and ATG16L1. In addition to that, there was a significant negative correlation between miRNA-106b and ATG16L1 (Table 3). This axis was predicted as a potential pathway for the anticancer effect of C3G on HCC (Figure 1).

### 3.4. Histopathological and Immunohistochemical Studies

**Figure 2:**
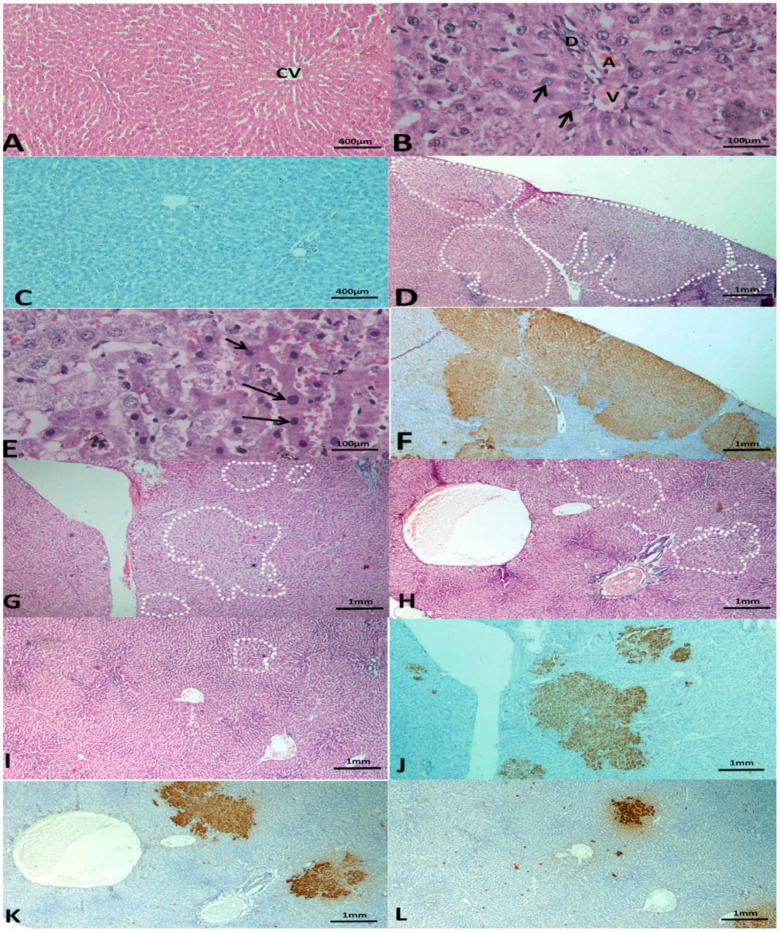
Photomicrographs of liver tissues after 112 days. (**A**) Normal control group with H & E stain showing normal hepatic architecture. (**B**) Normal control group with H & E stain showing polygonal hepatocytes (arrows) with central rounded vesicular nuclei, and a portal triad formed of a branch of the portal vein (V), bile ductile (D), and branch of the hepatic artery (A). (**C**) Normal control group with the immuno-histochemical stained section with Anti GST-P showing a negative reaction. (**A**) ×100 Hx & E stain, (**B**) ×400 Hx & E stain, (**C**) ×100 anti GST-P immuno-histochemical stain. (**D**) Group 2 treated with DEN and 2-AAF stained with H & E. Liver section showing larger, less discriminated dysplastic nodules compressing the surrounding liver tissue with disruption of normal hepatic lobular architecture (dotted shapes). (**E**) Group 2 treated with DEN and 2-AAF stained with H & E showing dysplastic alternating hepatocytes and dark hyper-chromatic nuclei and increased nuclear-cytoplasmic ratio (arrows). (**F**) Group 2 treated with DEN and 2-AAF with immuno-histochemical stained sections with GST-P antibody showing large multiple GST-P-positive hepatic nodules (brown stained) scattered in-between negatively stained hepatic parenchyma. (**D**) ×40 Hx & E stain, (**E**) ×400 Hx & E stain, (**F**) ×40 anti GST-P immuno-histochemical stain. (**G**) Treated group with C3G (10 mg/kg) stained with H & E, showing small and less discriminated dysplastic nodules (doted shapes). (**H**) Treated group with C3G (15 mg/kg) stained with H&E showing small and less discriminated dysplastic nodules (doted shapes). (**I**) Treated group with C3G (20 mg/kg) stained with H & E showing smaller and less discriminated dysplastic nodules (doted shapes) and the presence of autophagic vacuole (dotted arrows). (**J**) Treated group with C3G (10 mg/kg) with immuno-histochemical stained sections with GST-P antibody showing GST-P positive small hepatic foci (brown stain). (**K**) Treated group with C3G (15 mg/kg) with immuno-histochemical stained sections with GST-P antibody showing smaller GST-P positive hepatic foci (brown stain). (**L**) Treated group with C3G (20 mg/kg) with immuno-histochemical stained sections with GST-P antibody showing smaller GST-P positive hepatic foci (brown stain) and appearance of the autophagic vacuole (dotted arrows). (**G**–**I**) Hx & E stain and (**J**–**L**) anti-GST-P immuno-histochemical stain, magnification ×40. The number of rats per group equals six.

Livers of the normal control group had normal architecture; localised lesions, alternating pre-neoplastic foci, and dysplastic nodules were not observed (Figure 2A–C). Classic polygonal hepatocytes with central rounded vesicular nuclei and acidophilic granular cytoplasm were also observed. Portal triads were formed of a branch of the portal vein, bile ductile, and branch of the hepatic artery (Figure 2B). Livers of rats that received DEN + 2AAF showed the development of multiple aggregations of hepatocellular pre-neoplastic lesions. The lesions were large, less differentiated, and multiple dysplastic nodules appeared scattered and occupied most of the examined fields (Figure 2D–F). Nodules were formed of alternating hepatocytes showing dark hyper-chromatic nuclei and increased nuclear:cytoplasmic ratio (Figure 2E). Livers of rats treated with C3G (10, 15, and 20 mg/kg) had decreased number and size of lesions (Figure 2G–I). The effect was dose-dependent. The livers of rats treated with 10 mg/kg C3G showed small dysplastic nodules with no compression of the surrounding hepatic parenchyma. The livers of rats treated with 15 mg/kg and 20 mg/kg C3G had localised lesions in the form of alternating foci merging imperceptibly with hepatic tissue with no or minimal disruption of lobular architecture. Immunohistochemical staining with antibodies against GST-P was used to detect hepatic preneoplasia. Rats that received DEN + 2AAF had more positive GST-P nodules (Figure 2F). Injected groups with different C3G doses (10, 15, 20 mg/kg) exhibited a diminished number and size of the developed nodules (Figure 2J–L).

## 4. Discussion

HCC was estimated to be the second most prevalent form of cancer in Egypt after breast cancer [31]. In addition to that, HCC patients always suffer from recurrence and resistance against conventional chemotherapeutic agents [32]. Due to these reasons, the investigation of novel therapies or prophylactic agents for HCC is a major health priority. Most importantly, C3G showed promising, chemo-preventive activities against skin and lung cancer when it was tested on animal models [33]. C3G also showed anti-tumour action on ovarian cancer; this effect was attributed to the down-regulation of Mucin-4 protein [34].

Novel investigations recommended that activation of autophagy can promote programmed cell death in malignant tissue [35] and, consequently, suppress the tumour [36]. Consequently, the manipulation of autophagic signalling is an approach for the development of protective agents against resistant cancerous cells [37]. Delphinidin, a member of the anthocyanin group, induced growth retardation in HCC by promoting cellular vacuolisation [38]. In the current study, there was an improvement of the liver functions and histopathological profile in rats with precancerous lesions upon administration of C3G and an upregulation of the autophagy-related gene, ATG16L1.

ATG16L1, the current study target protein for expression analysis, is a member of the autophagy-related protein family with an activating role in the autophagy pathway; thus, it has a tumour-inhibitory role [39]. A study on breast cancer cells reported a high level of miRNA-20a that increase the reactive oxygen species and increase DNA damage by targeting autophagy activating proteins, including ATG16L1. This finding was consistent with the generated data of the current study, which also revealed that there was a significant negative correlation between miRNA-106b and ATG16L1. Here, in this study, administration of C3G had a chemoprotective effect on HCC with the upregulation of ATG16L1 and downregulation of miRNA106b.

To examine the relation between HCC and autophagy deficiency, P62 protein, which acts as a substrate and gets degraded in the autophagy process, was accumulated in HCC cells. At the same time, the adjacent non-tumour areas were negative for it [40]. Another study concerned with ATG genes, which are essential for the activation of autophagy, reported that mice with ATG7 knockout experienced the development of liver tumours [17]. ATG16L1 is a member of the autophagy-related protein family with an activating role in the autophagy pathway. It interacts with the complex molecular ATG12-ATG5 to form autophagosomes, fusing with lysosomes producing autolysosomes [41]. This protein was found to have a crucial defence role against invasive bacteria. At the site of bacterial entry, ATG16L1 was recruited to the plasma membrane through the effect of the intracellular sensors Nod1 and Nod2 [42].

Several tumours activating circRNAs have been reported. circRNA SMARCA5 regulates the progression of cervical cancer through inhibiting miRNA-432 [43], circRNA_0067934 promotes invasion of HCC through modulating the axis of miR-1324/FZD5/Wnt/β-catenin [44], circRNA LPAR3 sponges miRNA-198 causing upregulation of MET gene expression, which facilitates oesophagal cancer invasion [45], circRNA PRKCI inhibits miRNA-545 and induces glioma cell progression [46], and circRNA ZNF609 reduces miR-186-5p levels leading to induction of YAP1 and AMPK pathways and prostate cancer growth [47]. Inhibitory circRNAs include hsa_circ_0007059, which inactivates Wnt/β-catenin and ERK1/2 pathways due to inhibition of microRNA-378 leading to lung cancer suppression [48], circRNA C3P1 that inhibits HCC growth and metastasis through modulating miR-4641/PCK1 axis [49], circRNA MTO1 that up-regulates p21 expression and inhibits HCC invasion through sponging oncogenic miR-9 [50], and circRNA TRIM33-12 that also suppresses HCC through the inhibitory effect on miRNA-191 [51]. The involvement of circRNAs in regulatory networks make them potential biomarkers since they are stable, easily detected, and abundant in blood and saliva [52].

CircRNA acts as a sponge for miRNA on the molecular level [53]; therefore, they can have roles either as tumour activators or inhibitors according to the effect of miRNA on various cellular pathways and signalling. In this study, has_circ_0001345 had a tumour inhibitory role as it sponged miRNA106b. Overexpression of miRNA106b accelerated the epithelial–mesenchymal transition (EMT) process [54] and disrupted ATG16L1-mediated autophagy [55]. This is consistent with our results which showed that circRNA sponged miRNA106b leading to the upregulation of ATG16L1 upon administration with C3G.

Our findings encourage the development of an antimitotic approach that combines conventional cytotoxic medications with phytochemical extracts to prevent cancer cell cycle progression. Additional in vitro functional investigations are necessary to elucidate the RNA panel’s functional mechanism and to validate its significance as a drug target biomarker

## 5. Conclusions

Cyanidin-3-glucoside is a naturally occurring molecule that has the potential to aid in the future unravelling of the mystery of cytotoxic medicines. It has the potential to be exploited to develop novel antimitotic agents that specifically target the cell cycle in HCC. Additionally, our findings revealed that the hsa_circ_0001345/miRNA106b/ATG16L1 axis has significant promise as a therapeutic target biomarker for further research.

## Figures and Tables

**Table 1:** Liver functions and alfa fetoprotein (AFP)among the investigated groups of rats.

Groups	ALT (U/L)	Albumin (g/dL)	T Bilirubin (mg/dL)	D Bilirubin (mg/dL)	AFP (ng/mL)
Normal control	34 ± 2.52	3.67 ± 0.37	1.2 ± 0.11	0.66 ± 0.03	61.33 ± 13.93
PCL	224.1 ± 27.33 ^a^	1.76 ± 0.03 ^a^	3.43 ± 0.12 ^a^	2.83 ± 0.14 ^a^	2135.16 ± 588.08 ^a^
T1	107.5 ± 9.62 ^b^	2.7 ± 0.07 ^b^	2.35 ± 0.13 ^b^	1.60 ± 0.11 ^b^	266.21 ± 42.80 ^b^
T2	65.5 ± 5.4 b ^c^	2.81 ± 0.08 ^b^	1.72 ± 0.07 ^b,c^	1.20 ± 0.23 ^b^	160.16 ± 18.01 ^b^
T3	45.83 ± 1.66 ^b,c^	3.11 ± 0.15 ^b^	1.3 ± 0.09 ^b,c^	0.77 ± 0.06 ^b,c^	95.83 ± 11.36 ^b^

ALT, Alanine aminotransferase; T bilirubin, total bilirubin; D bilirubin, direct bilirubin; AFP, Alpha-fetoprotein; PCL, precancerous lesion group; T1, group treated with 10 mg/kg C3G; T2, group treated with 15 mg/kg C3G; and T3, group treated with 20 mg/kg C3G. Data were expressed as mean ± SD. The number of rats equals six for each group. ^a^ Significantly different from the normal control group, ^b^ Significantly different from the PCL group, ^c^ Significantly different from the T1 group. Specimen, serum.

**Table 2:** Expression levels (relative quantification, RQ) of circRNA 0001345, miRNA 106b, and mRNA ATG16L1 among the investigated groups of rats.

Groups	CircRNA 0001345	miRNA 106b	mRNA ATG16L1
	Fold Change Levels		
Normal control	0.91 ± 0.15	1.05 ± 0.20	1.46 ± 0.24
PCL	0.5 ± 0.11	22.34 ± 2.13 ^a^	0.08 ± 0.01
T1	2.67 ± 0.30	0.6 ± 0.08 ^b^	3.84 ± 0.42
T2	5.82 ± 0.61 ^b^	0.23 ± 0.06 ^b^	12.64 ± 1.76 ^b,c^
T3	23.37 ± 2.51 ^b,c,d^	0.21 ± 0.03 ^b^	19.66 ± 1.81 ^b,c,d^

PCL, precancerous lesion group; T1, group treated with 10 mg/kg C3G; T2, group treated with 15 mg/kg C3G; and T3, group treated with 20 mg/kg C3G. Data were expressed as mean ± SD. The number of rats equals six for each group. ^a^ significant difference from the normal control group, ^b^ significant difference from the PCL group, ^c^ significant difference from the T1 group, ^d^ a significant difference from the T2 group. Specimen, liver tissue.

**Table 3:** Correlation between competing endogenous RNAs (CircRNA 001345, miRNA 106b, and mRNA ATG16L1).

	CircRNA 001345		miRNA 106b		mRNA ATG16L1	
	r	*p* Value	r	*p* Value	r	*p* Value
CircRNA 001345			−0.326	0.12	0.767	<0.001 *
miRNA 106b	−0.326	0.12			−0.455	0.026 *

* Significantly correlated (correlation is significant at the 0.05 level).

## Data Availability

Available upon request from the corresponding author.
